# Diamine Oxidase Interactions with Anti-Inflammatory and Anti-Migraine Medicines in the Treatment of Migraine

**DOI:** 10.3390/jcm12237502

**Published:** 2023-12-04

**Authors:** Yaiza Tobajas, Marc Alemany-Fornés, Iris Samarra, Jordi Romero-Giménez, Maria Tintoré, Antoni del Pino, Núria Canela, Josep M. del Bas, Nàdia Ortega-Olivé, Carlos de Lecea, Xavier Escoté

**Affiliations:** 1 EURECAT, Centre Tecnològic de Catalunya, Nutrition and Health, 43204 Reus, Spain; yaiza.tobajas@eurecat.org (Y.T.); jordi.romerog@eurecat.org (J.R.-G.); nadia.ortega@eurecat.org (N.O.-O.); 2 DR Healthcare-AB Biotek HNH, 08017 Barcelona, Spain; malemany@dr-healthcare.com (M.A.-F.); maria.tintore@abbiotekhealth.com (M.T.); carlos.delecea@abbiotekhealth.com (C.d.L.); 3 Centre for Omic Sciences (COS), Joint Unit URV-EURECAT, Unique Scientific and Technical Infrastructures (ICTS), Eurecat, Centre Tecnològic de Catalunya, 43204 Reus, Spain; iris.samarra@eurecat.org (I.S.); antoni.delpino@eurecat.org (A.d.P.); nuria.canela@eurecat.org (N.C.); 4 EURECAT, Centre Tecnològic de Catalunya, Biotechnology Area, 43204 Reus, Spain; josep.delbas@eurecat.org; 5 Department of Biochemistry and Biotechnology, Universitat Rovira i Virgili, Campus Sescelades, 43007 Tarragona, Spain

**Keywords:** migraine, DAO, histamine intolerance, analgesics, NSAIDs, anti-inflammatory

## Abstract

Histamine intolerance arises when there is a disparity between the production of histamine and the body’s ability to break it down. In the gastrointestinal tract, the primary enzyme responsible for metabolizing ingested histamine is diamine oxidase (DAO), and a shortage of this enzyme has been associated with some diseases related to the respiratory, cardiovascular, nervous, muscular, and digestive systems, in addition to migraines. The treatment of migraines typically revolves around the utilization of both anti-migraine and anti-inflammatory drugs, but their interaction with DAO is not thoroughly understood. In this study, we examined the impact of nonsteroidal anti-inflammatory drugs (NSAIDs) and anti-migraine medications on DAO activity through in vitro experiments. We also investigated their effects on the human intestinal cell line Caco-2, assessing changes in DAO expression (both at the mRNA and protein levels) as well as DAO activity. The tested drugs, including ibuprofen, acetylsalicylic acid, paracetamol, a combination of acetylsalicylic acid with paracetamol and caffeine, zolmitriptan, and sumatriptan, did not inhibit DAO activity or reduce their levels. However, naproxen reduced DAO protein levels in human enterocyte cultures while not affecting DAO activity. These results suggest that combining anti-inflammatory and anti-migraine drugs with DAO enzyme supplementation for migraine patients with DAO deficiency could be beneficial for healthcare professionals in their daily practice.

## 1. Introduction

Headache disorders are on the rise worldwide, affecting billions of people in 2016 [1]. They include migraine and tension-type headaches, which contribute to disability-adjusted life years, and the global prevalence of headaches carries substantial societal costs and impacts [1]. Headaches are a common complaint worldwide, with primary headaches being much more common than secondary ones. Migraine and tension headaches are the most frequent primary headaches. [2]. Clinicians rely on the International Classification of Headache Disorders (ICHD) to diagnose and classify these conditions [3] aligned with the World Health Organization’s International Classification of Diseases (ICD-11) [4]. The management of migraine primarily involves the use of anti-migraine and anti-inflammatory drugs [5,6]. Different classes of medications are commonly employed for this purpose. Some analgesics, such as nonsteroidal anti-inflammatory drugs (NSAIDs) like ibuprofen and naproxen, are often recommended for their anti-inflammatory properties and pain-relieving effects [7,8]. These medications can help alleviate the intense pain and associated symptoms of migraines, providing relief to individuals experiencing these debilitating headaches [7]. Additionally, triptans, such as sumatriptan and rizatriptan, are widely used as specific anti-migraine drugs [9]. They work by constricting blood vessels in the brain and inhibiting the release of pain-inducing neurotransmitters [10]. Unfortunately, medications for migraine also have limitations in terms of efficacy, and many individuals continue to experience migraine attacks despite their use [11]. Additionally, long-term use of these medications may lead to undesirable side effects, further complicating treatment decisions [12]. Preventive treatment of episodic migraine includes nonspecific and migraine-specific drugs [13]. While medications from several pharmacological classes—such as anticonvulsants, beta-blockers, and antidepressants—have an established efficacy in migraine prevention, they are associated with a number of side effects [13]. Finally, medication-overuse headache is characterized by persistent headaches that occur due to the excessive use of various medications intended to relieve headaches [14]. The preferred treatment approach is discontinuing the overused medications. However, the current methods for achieving this withdrawal are primarily guided by expert recommendations rather than solid scientific evidence, partly because there is a lack of randomized controlled studies in this area.

Histamine is a neurotransmitter that plays a crucial role in various biological functions, including but not limited to inflammation, modulation of neural activity, and the control of immune responses [15]. The body naturally produces histamine, and it can also be obtained from dietary sources. However, when the levels of free histamine become elevated, it can lead to a condition called histamine intolerance (HIT), resulting in a range of undesirable symptoms including headaches, among others [16,17]. The primary mechanism for metabolizing diet histamine is in the gastrointestinal tract through the action of the diamine oxidase (DAO) enzyme and, consequently, deficiency in DAO is a major cause of HIT [18]. The International Society of DAO Deficiency reports that insufficient levels of DAO enzyme can lead to a range of negative impacts on different body systems [16]. These effects encompass the respiratory system (resulting in symptoms like nasal congestion and asthma [19]), the cardiovascular system (manifesting as hypotension, hypertension, and arrhythmias [20]), the central nervous system (causing conditions like hangover-like sensations, and dizziness [21]), the digestive system (contributing to issues such as irritable bowel syndrome, constipation, early satiety, stomach pain, and vomiting [22]), the muscular system (potentially leading to fibromyalgia and muscle pain [23]), and the skeletal system (potentially causing osteopathic pain [24]). In addition, HIT and DAO deficiency have been associated with migraines [25]. Indeed, single nucleotide polymorphisms (SNPs) in the DAO gene, linked with reduced activity of the DAO enzyme, are associated with the risk of developing migraine, particularly in women [26]. Although there is no definitive cure for HIT or DAO deficiency, individuals with these conditions can often manage their symptoms and enhance their quality of life through dietary adjustments and supplements like DAO enzymes [27]. Some pharmaceutical drugs, including proton pump inhibitors (PPIs) [28] and NSAIDs [29], have been reported to potentially inhibit DAO activity, which could lead to histamine-intolerance-related symptoms. Understanding these interactions is essential for patients with HIT who may be taking medications that affect DAO function.

Understanding the potential interactions between DAO and these medications is crucial because it could provide new insights into migraine management and may offer alternative strategies for improving migraine treatment, minimizing side effects, and reducing the risk of medication-overuse headaches. Thus, for the first time, this work evaluated the effects of DAO with the more common anti-inflammatory and anti-migraine drugs (ibuprofen, naproxen, acetyl salicylic, paracetamol, a commercially available combination of acetyl salicylic with paracetamol and caffeine -APC-, zolmitriptan, and sumatriptan). For this purpose, we conducted experiments to examine how DAO interacts with drugs used for anti-inflammatory and migraine treatment. We performed in vitro studies to directly test the inhibition of DAO activity. Additionally, we evaluated this interaction in a human gut intestinal cell line by examining changes in mRNA, protein levels, and enzyme activity.

## 2. Materials and Methods

The materials and methods are essentially explained in the study by Tobajas et al. [30], and they are briefly presented below. Details of Assessment of the Suppression of Diamine Oxidase Activity by Anti-Migraine Medications, Evaluation of Diamine Oxidase Activity Inhibition by Metabolized Anti-Migraine Medications, Liquid Chromatography-Tandem Mass Spectrometry (LC-MS/MS), In vitro cell culturing and experimental treatments, Isolation of RNA and quantitative polymerase chain reaction (RT-qPCR) analysis, Extraction of proteins; Western Blot analysis; DAO activity within Caco-2 enterocytes; Statistical methods are available in the Supplementary Materials (Supplementary Material S1, Figure S1).

### Cell Viability Measurement

Viability was measured by an MTT assay [2]. Cells were incubated for 24 h in the presence of three increased concentrations of the different treatments: ibuprofen (24, 194, and 388 µM); acetyl salicylic (2, 3.5, and 5 mM); paracetamol (10, 20, and 30 µM); APC (acetyl salicylic with paracetamol and caffeine; 150, 200, and 300 µM); naproxen (2.5, 3, and 5 µM); zolmitriptan (2, 3.5, and 5 µM); and sumatriptan (10, 20, and 100 µM). At the conclusion of the 24 h incubation period, the treatments were removed, and MTT was added to each well, incubating for additional 2 h at 37 °C. The resulting formazan precipitate was then resuspended in isopropanol. Absorbance readings were taken at both 570 nm and 650 nm as a reference. Viability values for the various treatments were calculated as a relative percentage compared to cells treated with the vehicle control. DMSO was employed as a negative viability control. Once the optimal dosage for each drug was determined, Caco-2 cells were seeded into 12-well plates to conduct the subsequent assays. These cells were exposed to the drugs of interest for 24 h to assess mRNA expression, protein expression, and DAO activity [30,31]. Furthermore, aminoguanidine was incorporated into the experimental design due to its established role as a DAO activity-inhibitory agent [32,33].

## 3. Results

### 3.1. DAO Activity Was Not Affected by the Presence of Anti-Inflammatory and Anti-Migraine Drugs

**Figure 1:**
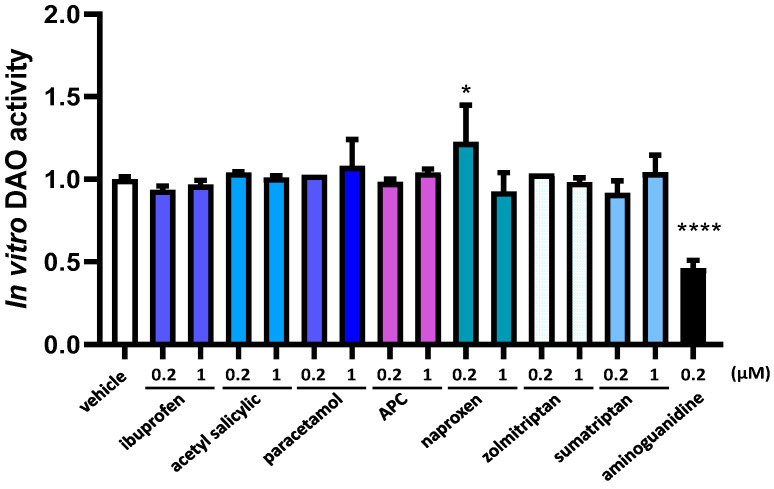
DAO activity was not affected by the presence of ibuprofen, acetyl salicylic, paracetamol, APC (acetyl salicylic with paracetamol and caffeine), naproxen, zolmitriptan, and sumatriptan at the indicated concentrations. In contrast, naproxen induced DAO activity at the lowest dose tested (0.2 µM) and aminoguanidine produced an important reduction in DAO activity. Data are expressed as mean ± SEM. The results are expressed relative to the vehicle group. One-way ANOVA test followed by Dunnett’s post hoc test, * *p* < 0.05; **** *p* < 0.0001 vs. vehicle.

LC-MS/MS chromatography in vitro analyses showed no effects of the presence acetyl salicylic, zolmitriptan, paracetamol, APC (acetyl salicylic with paracetamol and caffeine), ibuprofen, and sumatriptan on the reduction in DAO activity, nor in low and high concentrations (Figure 1). Surprisingly, naproxen promoted a higher DAO activity at a lower concentration (increase of 22%) whereas the positive control aminoguanidine induced a strong down-regulation in DAO activity (Figure 1).

**Figure 2:**
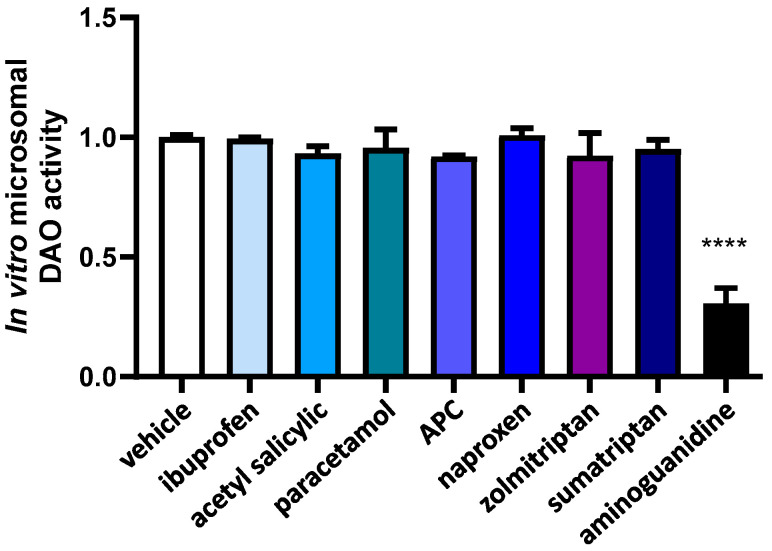
DAO activity was not affected by the presence ibuprofen, acetyl salicylic, paracetamol, APC (acetyl salicylic with paracetamol and caffeine), naproxen, zolmitriptan, nor sumatriptan previously incubated with microsomes. In contrast, aminoguanidine produced an important reduction in DAO activity. Data are expressed as mean ± SEM. The results are expressed relative to the vehicle group. One-way ANOVA test followed by Dunnett’s post hoc test, **** *p* < 0.0001 vs. vehicle.

In order to determine whether alterations in the chemical composition of anti-inflammatory and anti-migraine drugs that occur during hepatic metabolism can affect DAO activity, we exposed these compounds to hepatic microsomes before conducting DAO activity tests. (Figure 2). No effects in DAO activity were observed after the incubation with metabolized anti-inflammatory and anti-migraine drugs, whereas incubation with the positive control for inhibition, aminoguanidine, produced an important DAO activity repression.

### 3.2. Effects of Selected Anti-Inflammatory and Anti-Migraine Drugs on Intestinal DAO

**Figure 3:**
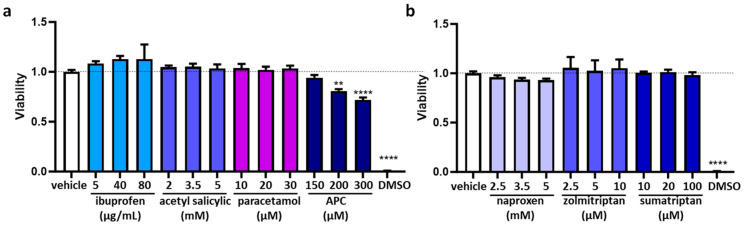
Cell viability in human enterocytes Caco-2 treated with the indicated concentrations of analgesics/anti-migraine drugs. (**a**) Ibuprofen, acetyl salicylic, paracetamol, and APC (acetyl salicylic with paracetamol and caffeine). (**b**) Naproxen, zolmitriptan, and sumatriptan. DMSO (dimethyl sulfoxide, 25%) was used as a negative control for viability. Data are expressed as mean ± SEM (*n* = 8). Results are expressed relative to the vehicle group. One-way ANOVA test followed by Dunnett’s post hoc test, ** *p* < 0.01, **** *p* < 0.0001 vs. vehicle.

To assess the concentrations of anti-inflammatory and anti-migraine drugs and their potential influence on DAO regulation in the human enterocytes Caco-2 cell line, we carried out cell viability tests (MTT assays) across a range of drug concentrations, spanning from low to moderate and high doses (Figure 3a,b). There were no notable differences between the effects of anti-inflammatory and anti-migraine drugs and those of the vehicle on the human enterocytes (Figure 3a,b). The exception was APC (acetyl salicylic with paracetamol and caffeine), which demonstrated toxicity when administered at medium and higher doses (Figure 3a). Consequently, the selected concentrations to carry out the following analysis were ibuprofen (388 µM); acetyl salicylic (5 mM); paracetamol (30 µM); APC (150 µM); naproxen (2.5 mM); zolmitriptan (10 µM); sumatriptan (20 µM); and aminoguanidine (10 mg/mL).

**Figure 4:**
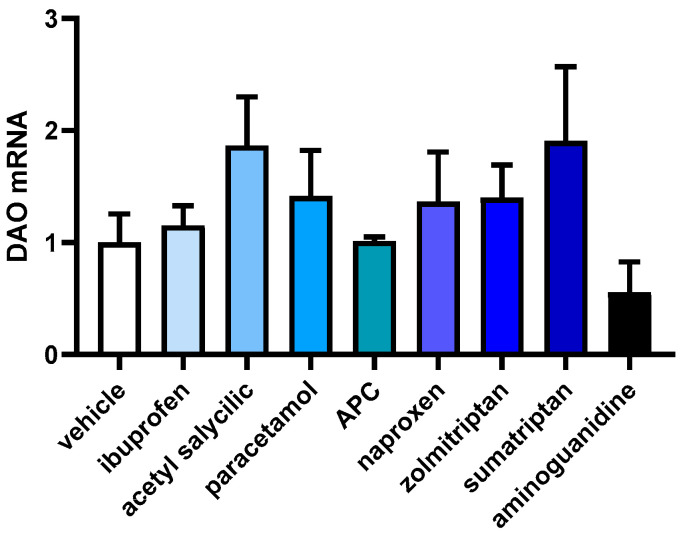
mRNA expression of DAO in human enterocytes Caco-2 treated with the analgesics/anti-migraine drugs (ibuprofen (388 µm); acetyl salicylic (5 Mm); paracetamol (30 µM); APC (acetyl salicylic with paracetamol and caffeine; 150 µM); naproxen (2.5 mM); zolmitriptan (10 µM), and sumatriptan (20 µM)); and aminoguanidine (10 mg/mL). Data are expressed as mean ± SEM (*n* = 3). The results are expressed relative to the vehicle group. One-way ANOVA test followed by Dunnett’s post hoc test.

In order to investigate how anti-inflammatory and anti-migraine drugs affect the gene expression of DAO in human Caco-2 enterocytes, mRNA expression assays (RT-qPCR) were performed (Figure 4). No differences were observed in the DAO mRNA expression enterocytes treated with the selected drugs, observing just a slight tendency to increase after the treatments with acetyl salicylic acid and sumatriptan. These results may suggest that anti-inflammatory and anti-migraine treatments do not interfere with DAO mRNA expression in human enterocytes.

**Figure 5:**
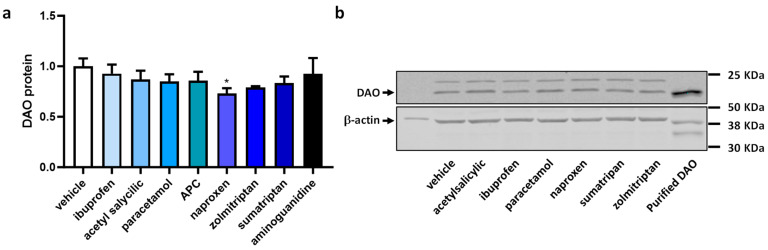
DAO protein expression in human enterocytes Caco-2 treated with the analgesics/anti-migraine drugs (ibuprofen (388 µM); acetyl salicylic (5 mM); paracetamol (30 µM); APC (acetyl salicylic with paracetamol and caffeine, 150 µM); naproxen (2.5 mM); zolmitriptan (10 µM); sumatriptan (20 µM)); and aminoguanidine (10 mg/mL). (**a**) Densitometry analysis of relative DAO protein concentration after the indicated treatments. (**b**) A representative Western blot analysis of human DAO and housekeeping β-actin levels. The results are expressed relative to the vehicle group. Data are expressed as mean ± SEM (*n* = 3). One-way ANOVA test followed by Dunnett post hoc test, * *p* < 0.05 vs. vehicle.

To assess the impact of anti-inflammatory and anti-migraine drugs on the protein expression of DAO in human Caco-2 enterocytes, protein expression assays (Western blot) were performed (Figure 5). Aminoguanidine was employed as a negative regulator for DAO activity. Protein expression of DAO did not change in enterocytes treated with anti-inflammatory and anti-migraine drugs, with the exception of naproxen, which showed an important reduction in the DAO protein levels (Figure 5). These findings could indicate that treatments aimed at reducing inflammation and addressing migraines may not have a substantial impact on the regulation of intracellular DAO protein levels in human enterocytes.

**Figure 6:**
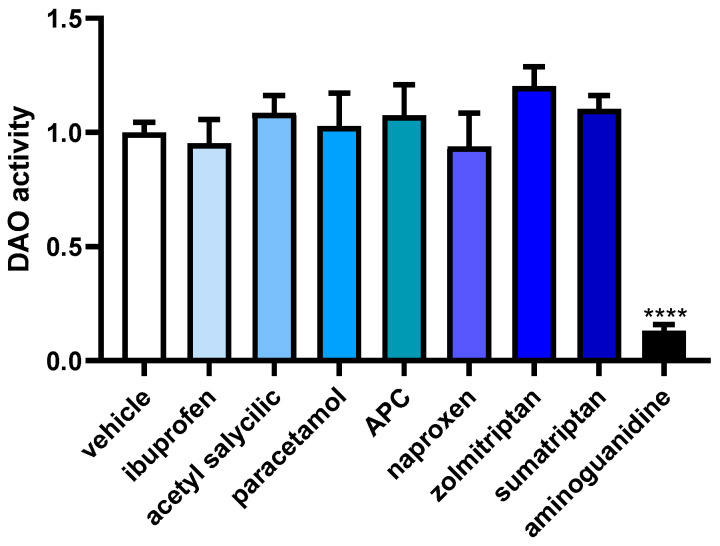
DAO activity in human enterocytes Caco-2 treated with the analgesic/anti-migraine drugs (ibuprofen (388 µM); acetyl salicylic (5 mM); paracetamol (30 µM); APC (acetyl salicylic with paracetamol and caffeine; 150 µM); naproxen (2.5 mM); zolmitriptan (10 µM), and sumatriptan (20 µM)); and aminoguanidine (10 mg/mL). Data are expressed as mean ± SEM (*n* = 3). The results are expressed relative to the vehicle group and relative to the amount of protein added in the assay. One-way ANOVA test followed by Dunnett post hoc test, **** *p* < 0.0001 vs. vehicle.

To assess how anti-inflammatory and anti-migraine drugs influence DAO activity in human Caco-2 enterocytes, we conducted a specialized DAO fluorescent activity assay (Figure 6)**.** No significant differences were observed for any of the anti-inflammatory and anti-migraine tested drugs. As described in the literature [32], a strong down-regulation in the DAO activity was observed in enterocytes treated with the aminoguanidine. These findings may indicate that anti-inflammatory and anti-migraine medicines do not modulate DAO activity in human enterocytes.

## 4. Discussion

The present research supports that the standard therapies for migraine treatment do not disrupt the function of DAO. This was confirmed through the assessment of DAO activity in both laboratory-based experiments as well as in a human cell line of intestinal epithelium. It is important to have in mind that naproxen was the unique medication that displayed a decrease in the expression of DAO protein. However, this reduction in protein levels did not result in a corresponding decrease in DAO activity, implying a lingering adverse impact of naproxen on the DAO system. Results of this study are relevant because the prevalence of migraine is increasing in various countries, regardless of gender, ethnicity, or economic conditions [34,35] and, consequently, it is highly probable that migraine patients present HIT or DAO deficiency. Based on previous clinical evidence [18], DAO supplementation could be a promising strategy in migraine patients with DAO deficiency, compatible with current pharmacological protocols. However, it is important to note that the specific interactions can vary depending on the individual and the pharmaceutical drug. Thus, healthcare providers should consider these interactions when prescribing drugs to treat migraine to individuals with HIT or DAO deficiency and carefully monitor their response to these treatments. These beneficial effects of DAO supplementation may be applied in other circumstances, such as in the management of medication overuse headache, which is a chronic secondary headache that results from the overuse of medication [14,36]. This is a prevalent clinical issue that necessitates effective management due to the substantial impairment experienced by these patients [36]. The best treatment strategy for medication overuse headache has been debated for years [37,38]; nonetheless, incorporating DAO supplementation as a complementary approach in patients with HIT or DAO deficiency could potentially assist in alleviating the symptoms associated with medication-overuse headaches. Similarly, the addition of DAO supplementation may lead to a decrease in the necessity, dosage, or duration of anti-migraine medications to address symptoms, consequently lowering the risk of experiencing adverse side effects [39]. This novel adjuvant role of DAO supplementation in migraine treatment has the potential to decrease the frequency and severity of migraine episodes, which, in turn, may reduce the chronic reliance on these medications [18], reserving their use for specific rescue situations when necessary. Hence, DAO supplementation might become the favoured choice for migraine prevention in individuals with HIT or DAO deficiency. In addition a higher hypersensitivity to NSAIDs in patients carrying an SNP of DAO, which causes decreased DAO metabolic capacity, has been described [40], which represents an additional limitation in the management of migraine in these group pf patients. DAO supplementation should complement physiological processes and could lead to a reduced reliance on other preventive medications or the doses required to achieve their preventive benefits.

Although a reduction in DAO protein expression was detected after exposure to naproxen, this alteration did not have a substantial impact on DAO activity. This finding illustrates that the recommended DAO dosage might require adjustments when taking it with naproxen and could potentially explain the lack of treatment effects or even exacerbated migraine symptoms. This is because naproxen could further reduce the ability to metabolize histamine in patients with DAO deficiency, triggering a transient HIT or DAO deficiency in migraine patients without an underlying primary DAO deficiency as a result of drug interactions [41]. This negative effect could be a consequence of the action of non-coding microRNAs (e.g., miR-34a-5p and miR-375) as regulators of DAO and, consequently, neuroinflammation, which is of great importance in adolescents who use naproxen [42,43,44].

There are several limitations of this study. One limitation of the current study is that the behaviour of cultured cells grown in a lab setting may not accurately mirror how cells behave within a living organism without capturing the natural variability observed in individuals’ responses to treatments or interventions, which restricts the applicability of our findings to humans. This discrepancy arises because cells in living organisms interact with various other cells, the extracellular matrix, immune system interactions, and the surrounding environment in intricate and ever-changing ways that are challenging to replicate in a laboratory. Furthermore, cells cultivated in a lab environment may undergo alterations in gene expression or behaviour due to the artificial conditions of their culture, including the absence of natural physical forces or the presence of growth factors that differ from those found in living organisms. To address these limitations, future research could explore preclinical animal models that mimic the complex microenvironment of living organisms more closely and allow for the evaluation of long-term effects and chronic conditions. Moreover, the results obtained from this study should be confirmed through clinical trials involving migraine patients.

## 5. Conclusions

To sum up, the findings from the selected anti-inflammatory and anti-migraine drugs, including acetylsalicylic acid, zolmitriptan, paracetamol, a combination of acetylsalicylic acid with paracetamol and caffeine, ibuprofen, and sumatriptan, did not demonstrate any inhibitory effects on the activity or levels of DAO, both in in vitro experiments and in human enterocytes cultures. In contrast, exposure to naproxen resulted in a decrease in DAO protein levels in human enterocyte cultures, without affecting DAO activity. Interestingly, in in vitro assays, naproxen even led to an increased DAO activity. Based on these findings, it appears that prescribing a combination of anti-inflammatory and anti-migraine medications alongside DAO enzyme supplementation for migraine patients who also have DAO deficiency may represent a valuable strategy in the management of migraine and in the prevention of medication-overuse headaches.

## Data Availability

The data presented in this study are available on request from the corresponding author.

## Supplementary Materials

The following supporting information can be downloaded at: https://www.mdpi.com/article/10.3390/jcm12237502/s1, Supplementary Material S1: Supplementary Materials and Methods; Figure S1: The analysis of DAO protein levels using Western Blot. References [45,46,47,48,49,50,51,52,53,54,55,56,57,58,59,60] are cited in the Supplementary Materials.

## References

[1] Stovner L.J., Nichols E., Steiner T.J., Abd-Allah F., Abdelalim A., Al-Raddadi R.M., Ansha M.G., Barac A., Bensenor I.M., Doan L.P. (2018). Global, regional, and national burden of migraine and tension-type headache, 1990–2016: A systematic analysis for the Global Burden of Disease Study 2016. Lancet Neurol..

[2] Stovner L.J., Hagen K., Linde M., Steiner T.J. (2022). The global prevalence of headache: An update, with analysis of the influences of methodological factors on prevalence estimates. J. Headache Pain.

[3] Kim B.-K., Cho S.-J., Kim B.-S., Sohn J.-H., Kim S.-K., Cha M.-J., Song T.-J., Kim J.-M., Park J.W., Chu M.K. (2016). Comprehensive Application of the International Classification of Headache Disorders Third Edition, Beta Version. J. Korean Med. Sci..

[4] Headache Classification Committee of the International Headache Society (IHS) (2013). The International Classification of Headache Disorders, 3rd edition (beta version). Cephalalgia.

[5] Ng J.Y., Hanna C. (2021). Headache and migraine clinical practice guidelines: A systematic review and assessment of complementary and alternative medicine recommendations. BMC Complement. Med. Ther..

[6] Eigenbrodt A.K., Ashina H., Khan S., Diener H.-C., Mitsikostas D.D., Sinclair A.J., Pozo-Rosich P., Martelletti P., Ducros A., Lantéri-Minet M. (2021). Diagnosis and management of migraine in ten steps. Nat. Rev. Neurol..

[7] VanderPluym J.H., Halker Singh R.B., Urtecho M., Morrow A.S., Nayfeh T., Torres Roldan V.D., Farah M.H., Hasan B., Saadi S., Shah S. (2021). Acute Treatments for Episodic Migraine in Adults: A Systematic Review and Meta-analysis. JAMA.

[8] Mayans L., Walling A. (2018). Acute Migraine Headache: Treatment Strategies. Am. Fam. Physician.

[9] Marmura M.J., Silberstein S.D., Schwedt T.J. (2015). The acute treatment of migraine in adults: The american headache society evidence assessment of migraine pharmacotherapies. Headache.

[10] Nicolas S., Nicolas D. (2023). Triptans.

[11] Ong J.J.Y., De Felice M. (2018). Migraine Treatment: Current Acute Medications and Their Potential Mechanisms of Action. Neurother. J. Am. Soc. Exp. Neurother..

[12] Berger A.A., Winnick A., Popovsky D., Kaneb A., Berardino K., Kaye A.M., Cornett E.M., Kaye A.D., Viswanath O., Urits I. (2020). Lasmiditan for the Treatment of Migraines with or without Aura in Adults. Psychopharmacol. Bull..

[13] Chua A.L., Mehla S., Orlova Y.Y. (2022). Drug Safety in Episodic Migraine Management in Adults. Part 2: Preventive Treatments. Curr. Pain Headache Rep..

[14] Kristoffersen E.S., Lundqvist C. (2014). Medication-overuse headache: Epidemiology, diagnosis and treatment. Ther. Adv. Drug Saf..

[15] Branco A.C.C.C., Yoshikawa F.S.Y., Pietrobon A.J., Sato M.N. (2018). Role of Histamine in Modulating the Immune Response and Inflammation. Mediat. Inflamm..

[16] Maintz L., Novak N. (2007). Histamine and histamine intolerance. Am. J. Clin. Nutr..

[17] Comas-Basté O., Sánchez-Pérez S., Veciana-Nogués M.T., Latorre-Moratalla M., Vidal-Carou M.D.C. (2020). Histamine Intolerance: The Current State of the Art. Biomolecules.

[18] Izquierdo-Casas J., Comas-Basté O., Latorre-Moratalla M.L., Lorente-Gascón M., Duelo A., Soler-Singla L., Vidal-Carou M.C. (2019). Diamine oxidase (DAO) supplement reduces headache in episodic migraine patients with DAO deficiency: A randomized double-blind trial. Clin. Nutr..

[19] Mayo-Yáñez M., Díaz-Díaz A., Vázquez-Barro J.C., Herranz González-Botas J., Figueroa A., Martín-Martín C.S. (2021). Relationship between allergic rhinitis and diamine oxidase activity: A preliminary report. Allergol. Sel..

[20] Zhao Y., Zhang X., Jin H., Chen L., Ji J., Zhang Z. (2022). Histamine Intolerance-A Kind of Pseudoallergic Reaction. Biomolecules.

[21] Cucca V., Ramirez G.A., Pignatti P., Asperti C., Russo M., Della-Torre E., Breda D., Burastero S.E., Dagna L., Yacoub M.-R. (2022). Basal Serum Diamine Oxidase Levels as a Biomarker of Histamine Intolerance: A Retrospective Cohort Study. Nutrients.

[22] Schnedl W.J., Lackner S., Enko D., Schenk M., Holasek S.J., Mangge H. (2019). Evaluation of symptoms and symptom combinations in histamine intolerance. Intest. Res..

[23] Okutan G., Ruiz Casares E., Perucho Alcalde T., Sánchez Niño G.M., Penadés B.F., Terrén Lora A., Torrente Estríngana L., López Oliva S., San Mauro Martín I. (2023). Prevalence of Genetic Diamine Oxidase (DAO) Deficiency in Female Patients with Fibromyalgia in Spain. Biomedicines.

[24] Silva A.C.d.O., Biasotto-Gonzalez D.A., Oliveira F.H.M., Andrade A.O., Gomes C.A.F., de P., Lanza F., de C., Amorim C.F., Politti F. (2018). Effect of Osteopathic Visceral Manipulation on Pain, Cervical Range of Motion, and Upper Trapezius Muscle Activity in Patients with Chronic Nonspecific Neck Pain and Functional Dyspepsia: A Randomized, Double-Blind, Placebo-Controlled Pilot Study. Evid. Based Complement. Alternat. Med..

[25] Schnedl W.J., Enko D. (2021). Histamine Intolerance Originates in the Gut. Nutrients.

[26] García-Martín E., Martínez C., Serrador M., Alonso-Navarro H., Ayuso P., Navacerrada F., Agúndez J.A.G., Jiménez-Jiménez F.J. (2015). Diamine oxidase rs10156191 and rs2052129 variants are associated with the risk for migraine. Headache.

[27] Schnedl W.J., Schenk M., Lackner S., Enko D., Mangge H., Forster F. (2019). Diamine oxidase supplementation improves symptoms in patients with histamine intolerance. Food Sci. Biotechnol..

[28] Siletsky S.A. (2023). Proton Pumps: Molecular Mechanisms, Inhibitors and Activators of Proton Pumping. Int. J. Mol. Sci..

[29] Leitner R., Zoernpfenning E., Missbichler A. (2014). Evaluation of the inhibitory effect of various drugs / active ingredients on the activity of human diamine oxidase in vitro. Clin. Transl. Allergy.

[30] Tobajas Y., Alemany-Fornés M., Samarra I., Romero-Giménez J., Tintoré M., Del Pino A., Canela N., Del Bas J.M., Ortega-Olivé N., de Lecea C. (2023). Interaction of Diamine Oxidase with Psychostimulant Drugs for ADHD Management. J. Clin. Med..

[31] Mettler L.G., Brecht K., Butterweck V., Meyer Zu Schwabedissen H.E. (2022). Impact of the clinically approved Petasites hybridus extract Ze 339 on intestinal mechanisms involved in the handling of histamine. Biomed. Pharmacother..

[32] Rokkas T., Vaja S., Murphy G.M., Dowling R.H. (1990). Aminoguanidine blocks intestinal diamine oxidase (DAO) activity and enhances the intestinal adaptive response to resection in the rat. Digestion.

[33] Yang R., Chen H., Gu Z. (2011). Factors influencing diamine oxidase activity and γ-aminobutyric acid content of fava bean (*Vicia faba* L.) during germination. J. Agric. Food Chem..

[34] Amiri P., Kazeminasab S., Nejadghaderi S.A., Mohammadinasab R., Pourfathi H., Araj-Khodaei M., Sullman M.J.M., Kolahi A.-A., Safiri S. (2021). Migraine: A Review on Its History, Global Epidemiology, Risk Factors, and Comorbidities. Front. Neurol..

[35] Stewart W.F., Roy J., Lipton R.B. (2013). Migraine prevalence, socioeconomic status, and social causation. Neurology.

[36] Carlsen L.N., Rouw C., Westergaard M.L., Nielsen M., Munksgaard S.B., Bendtsen L., Jensen R.H. (2021). Treatment of medication overuse headache: Effect and predictors after 1 year-A randomized controlled trial. Headache.

[37] Lipton R.B. (2015). Risk Factors for and Management of Medication-Overuse Headache. Contin. Lifelong Learn. Neurol..

[38] Diener H.-C., Dodick D., Evers S., Holle D., Jensen R.H., Lipton R.B., Porreca F., Silberstein S., Schwedt T. (2019). Pathophysiology, prevention, and treatment of medication overuse headache. Lancet Neurol..

[39] González-Hernández A., Marichal-Cancino B.A., MaassenVanDenBrink A., Villalón C.M. (2018). Side effects associated with current and prospective antimigraine pharmacotherapies. Expert Opin. Drug Metab. Toxicol..

[40] Agúndez J.A.G., Ayuso P., Cornejo-García J.A., Blanca M., Torres M.J., Doña I., Salas M., Blanca-López N., Canto G., Rondon C. (2012). The diamine oxidase gene is associated with hypersensitivity response to non-steroidal anti-inflammatory drugs. PLoS ONE.

[41] Manzotti G., Breda D., Di Gioacchino M., Burastero S.E. (2016). Serum diamine oxidase activity in patients with histamine intolerance. Int. J. Immunopathol. Pharmacol..

[42] Oskoui M., Pringsheim T., Holler-Managan Y., Potrebic S., Billinghurst L., Gloss D., Hershey A.D., Licking N., Sowell M., Victorio M.C. (2019). Practice guideline update summary: Acute treatment of migraine in children and adolescents: Report of the Guideline Development, Dissemination, and Implementation Subcommittee of the American Academy of Neurology and the American Headache Society. Neurology.

[43] Gallelli L., Cione E., Peltrone F., Siviglia S., Verano A., Chirchiglia D., Zampogna S., Guidetti V., Sammartino L., Montana A. (2019). Hsa-miR-34a-5p and hsa-miR-375 as Biomarkers for Monitoring the Effects of Drug Treatment for Migraine Pain in Children and Adolescents: A Pilot Study. J. Clin. Med..

[44] Gallardo V.J., Gómez-Galván J.B., Asskour L., Torres-Ferrús M., Alpuente A., Caronna E., Pozo-Rosich P. (2023). A study of differential microRNA expression profile in migraine: The microMIG exploratory study. J. Headache Pain.

[45] Comas-Basté O., Latorre-Moratalla M.L., Sánchez-Pérez S., Veciana-Nogués M.T., Vidal-Carou M.C. (2019). In vitro determination of diamine oxidase activity in food matrices by an enzymatic assay coupled to UHPLC-FL. Anal. Bioanal. Chem..

[46] Knights K.M., Stresser D.M., Miners J.O., Crespi C.L. (2016). In Vitro Drug Metabolism Using Liver Microsomes. Curr. Protoc. Pharmacol..

[47] Yoshitomo A., Asano S., Hozuki S., Tamemoto Y., Shibata Y., Hashimoto N., Takahashi K., Sasaki Y., Ozawa N., Kageyama M. (2022). Significance of Basal Membrane Permeability of Epithelial Cells in Predicting Intestinal Drug Absorption. Drug Metab. Dispos..

[48] Wu Q.Y., Ma S.Z., Zhang W.W., Yao K.B., Chen L., Zhao F., Zhuang Y.Q. (2018). Accumulating pathways of γ-aminobutyric acid during anaerobic and aerobic sequential incubations in fresh tea leaves. Food Chem..

[49] Guo X.-X., Zeng Z., Qian Y.-Z., Qiu J., Wang K., Wang Y., Ji B.-P., Zhou F. (2019). Wheat Flour, Enriched with γ-Oryzanol, Phytosterol, and Ferulic Acid, Alleviates Lipid and Glucose Metabolism in High-Fat-Fructose-Fed Rats. Nutrients.

[50] Hao H., Wang G., Sun J., Ding Z., Wu X., Roberts M. (2005). Unidirectional Inversion of Ibuprofen in Caco-2 Cells: Developing a Suitable Model for Presystemic Chiral Inversion Study. Biol. Pharm. Bull..

[51] Yu L.S., Zhao N.P., Yao T.W., Zeng S. (2006). Zolmitriptan uptake by human intestinal epithelial Caco-2 cells. Die Pharm. -Int. J. Pharm. Sci..

[52] Stevenson C.L., Augustijns P.F., Hendren R. (1999). Use of Caco-2 cells and LC/MS/MS to screen a peptide combinatorial library for permeable structures. Int. J. Pharm..

[53] Durham P.L., Russo A.F. (1999). Regulation of Calcitonin Gene-Related Peptide Secretion by a Serotonergic Antimigraine Drug. J. Neurosci..

[54] Siissalo S., Laine L., Tolonen A., Kaukonen A.M., Finel M., Hirvonen J. (2010). Caco-2 cell monolayers as a tool to study simultaneous phase II metabolism and metabolite efflux of indomethacin, paracetamol and 1-naphthol. Int. J. Pharm..

[55] Ricchi P., Di Palma A., Di Matola T., Apicella A., Fortunato R., Zarrilli R., Acquaviva A.M. (2003). Aspirin Protects Caco-2 Cells from Apoptosis after Serum Deprivation through the Activation of a Phosphatidylinositol 3-Kinase/AKT/p21*^Cip^*^/***WAF*1**^Pathway. Mol. Pharmacol..

[56] Kulthong K., Duivenvoorde L., Sun H., Confederat S., Wu J., Spenkelink B., de Haan L., Marin V., van der Zande M., Bouwmeester H. (2020). Microfluidic chip for culturing intestinal epithelial cell layers: Characterization and comparison of drug transport between dynamic and static models. Toxicol. Vitr..

[57] Jagannath V., Marinova Z., Monoranu C.-M., Walitza S., Grünblatt E. (2017). Expression of D-Amino Acid Oxidase (DAO/DAAO) and D-Amino Acid Oxidase Activator (DAOA/G72) during Development and Aging in the Human Post-mortem Brain. Front. Neuroanat..

[58] Panina Y., Germond A., Masui S., Watanabe T.M. (2018). Validation of Common Housekeeping Genes as Reference for qPCR Gene Expression Analysis During iPS Reprogramming Process. Sci. Rep..

[59] Quesada-Vázquez S., Colom-Pellicer M., Navarro-Masip È., Aragonès G., Del Bas J.M., Caimari A., Escoté X. (2021). Supplementation with a Specific Combination of Metabolic Cofactors Ameliorates Non-Alcoholic Fatty Liver Disease, Hepatic Fibrosis, and Insulin Resistance in Mice. Nutrients.

[60] Beltrán-Ortiz C., Peralta T., Ramos V., Durán M., Behrens C., Maureira D., Guzmán M.A., Bastias C., Ferrer P. (2020). Standardization of a colorimetric technique for determination of enzymatic activity of diamine oxidase (DAO) and its application in patients with clinical diagnosis of histamine intolerance. World Allergy Organ. J..

