# Supplementary material for: Diamine Oxidase Interactions with Anti-Inflammatory and Anti-Migraine Medicines in the Treatment of Migraine
Source: J Clin Med. 2023 Dec 4;12(23):7502. doi: 10.3390/jcm12237502 (PMC10707353; doi:10.3390/jcm12237502)
Supplement: Supplementary file 1 [file jcm-12-07502-s001.zip › jcm-2674429-supplementary.pdf]

Supplementary Material S1. Supplementary Materials and Methods.

The materials and methods are essentially explained in the study by Tobajas et al. [30]. and they will be briefly presented below:

Assessment of the Suppression of Diamine Oxidase Activity by Anti-Migraine Medications.

Each of the anti-inflammatory and anti-migraine drugs were prepared and were mixed with DAO (0.1 mg/mL) in phosphate buffer solution (PBS) at two final drug concentration (1 µM and 0.2 µM). Samples were placed in a water bath (1 h, 37 °C, 40 U/min) and 0.5 mL of 9 mM histamine were added to the sample. All the samples were kept in constant incubation, obtaining aliquots at different sampling times (t = 0, 0.5, 1, 1.5, 2, and 3 h) [45]. Perchloric acid was added to stop the enzymatic reaction. Internal standard (Histamine-d4) was also added. Samples were mixed and centrifuged at 15,000 rpm and the supernatant was diluted in 0.1 % formic acid in acetonitrile and transferred to vials for LC-MS/MS analysis. Two control samples were performed with DAO (0.1 mg/mL) and aminoguanidine (20 nM) in a DAO solution.

Evaluation of Diamine Oxidase Activity Inhibition by Metabolized Anti-Migraine Medications.

To understand whether modifications of acetyl salicylic, zolmitriptan, paracetamol, APC, ibuprofen, sumatriptan and naproxen produced effects on DAO inhibition, anti-inflammatory and anti-migraine drugs were treated with hepatic microsomes to simulate the metabolization of them by the liver [46]. For this, each of the anti-inflammatory and anti-migraine drugs (ibuprofen 0.225 µM; acetyl salicylic 0.8 µM; paracetamol 1 µM; APC 1 µM; naproxen 1 µM; zolmitriptan 0.225 µM; and sumatriptan 1 µM) were incubated with microsomes (20 mg/mL; Fisher Scientific). The reaction was started with the addition of NADPH, incubating the samples for 1 h (37 °C, 40 U/min). Samples were centrifuged and the supernatant was used as the "test drug" for the enzymatic assay. The enzymatic reaction was started, incubated, sampling times, stopped and transferred to glass vials for LC-MS/MS analysis as explained in the *Analysis of DAO activity inhibition* section. Internal standard (Histamine-d4) was also incorporated [45].

Liquid Chromatography-Tandem Mass Spectrometry (LC-MS/MS)

The chromatographic separation was performed with a gradient detailed in the study by Tobajas et al. [30]. Mobile phase was water with ammonium formate and acetonitrile. The column temperature was set at 45 ºC. The source parameters applied operating in positive electrospray ionization (ESI+) are explained in the study by Tobajas et al. [30]. The MRM transitions used as well as the retention time for each compound are summarized in the methods section explained by Tobajas et al. [30]. The absolute value of the slope of the histamine consumption (30–120 min) represented in nmol was used to determine DAO activity in mU (nmol/min) and expressed relative to the vehicle group.

***In vitro* cell culturing and experimental treatments.**

The human colonic epithelial cell line Caco-2 (American Type Culture Collection, ATCC) was maintained at standard culture conditions in Dulbecco's Modified Eagle Medium (DMEM) containing glucose, inactivated foetal bovine serum (FBS), non-essential amino acids and penicillin–streptomycin. For all the experiments, cells were maintained for 14 days until enterocytes were completely differentiated [47–49]. Based on the concentration ranges for anti-inflammatory and anti-migraine drugs described in the literature [50–56] it was prepared a stock solution to treat the enterocytes considering the molecular weight, the dose of the active compound and the weight of a pill [30]. The pills were ground into a fine powder and powder drugs were solubilized with dimethyl sulfoxide (DMSO) as a carrier. Stock solutions were filtered and were stored until they use.

Isolation of RNA and Quantitative Polymerase Chain Reaction (RT-qPCR) analysis

Caco-2 homogenates were used for total RNA extractions using TriPure reagent (Roche Diagnostic, Barcelona, Spain) [30]. RNA concentration and purity were determined using a nanophotometer (Implen GmbH, München, Germany) and retrotranscribed to cDNA using the High-Capacity RNA-to-cDNA Kit (Applied Biosystems, Wilmington, DE, USA). The cDNAs were diluted before incubation with commercial LightCycler 480 Sybr green I master on a Lightcycler® 480 II (Roche Diagnostic). Primers were previously described in other studies and verified with Primer-Blast software (National Center for Biotechnology Information, Bethesda, MD, USA). Sequences of oligonucleotides used in this study were: DAO F1: 5'-CGCAGACGTGATTGTCAACT-3'; DAO R1 5'-GGATGATGTACGGGGAATTG-3' [57]; PGK1 F1: 5'-CAAGAAGTATCTGTCA-3'; PGK1 R1: 5'-CGAAGGTGGAAGAGTGGGAGTTG-3' [58].

Extraction of proteins and Western Blot analysis.

Fully differentiated enterocytes were treated for 24 h with the selected drugs and homogenized with lysis buffer (NaH_2PO_4, Na_2HPO_4, SDS, NaCl, NP40, NaF, sodium orthovanadate, PMSF, and protease inhibitor cocktail 1 (Millipore Sigma, Germany)) [30]. The protein extracts were quantified by the BCA method (Bio-Rad Protein Assay; BioRad, CA, USA). 25 μg of protein extracts were separated on SDS-PAGE and electroblotted to nitrocellulose membranes (LI-COR Biosciences, NE, USA) [30]. Efficient protein transfer was monitored by Ponceau-S stain. Next, membranes were blocked (5% BSA) and probed with primary antibodies against DAO (PA5-76708, Invitrogen, Carlsbad, CA, USA) and β-Actin (Santa Cruz Biotechnology, Inc.; TX, USA). Then, infrared fluorescent secondary antibodies anti-rabbit 680, anti-rabbit 800 and anti-mouse 680 (LI-COR Biosciences; 926-32211, 926-68071 and 926-68070, respectively) were used for detection and quantified using ImageJ [59].

DAO activity within Caco-2 enterocytes

DAO activity was measured with a Diamine Oxidase Activity Assay Kit (Sigma-Aldrich; Germany) [60]. Briefly, this assay provides a straightforward method to determine DAO activity of Caco-2 cell lysates. In the assay, DAO converts the provided substrate, yielding an intermediate and hydrogen peroxide (H_2O_2). H_2O_2 is then utilized by the DAO Enzyme Mix to generate fluorescence (excitation: 535 nm/emission: 587 nm) from the DAO Probe. Finally, the DAO activity was expressed relative to the amount of protein added in the assay. Thus, an aliquot of the enterocytes extracts was quantified by BCA.

Statistical analysis

For each determination, an initial exploration was carried out to rule out discrepant points within the groups. For this purpose, the Grubbs statistical test was used, using the GraphPad Prism 10 software (GraphPad Software, Inc., La Jolla, USA) [59]. All data are expressed as means ± standard error of the mean (SEM). To analyse differences in the different parameters, a one-way variability analysis (One Way ANOVA) was used followed by Dunnett post-hoc test was performed to analyse differences of every treatment to a vehicle mean. A probability level of $p < 0.05$ was defined as statistically significant.

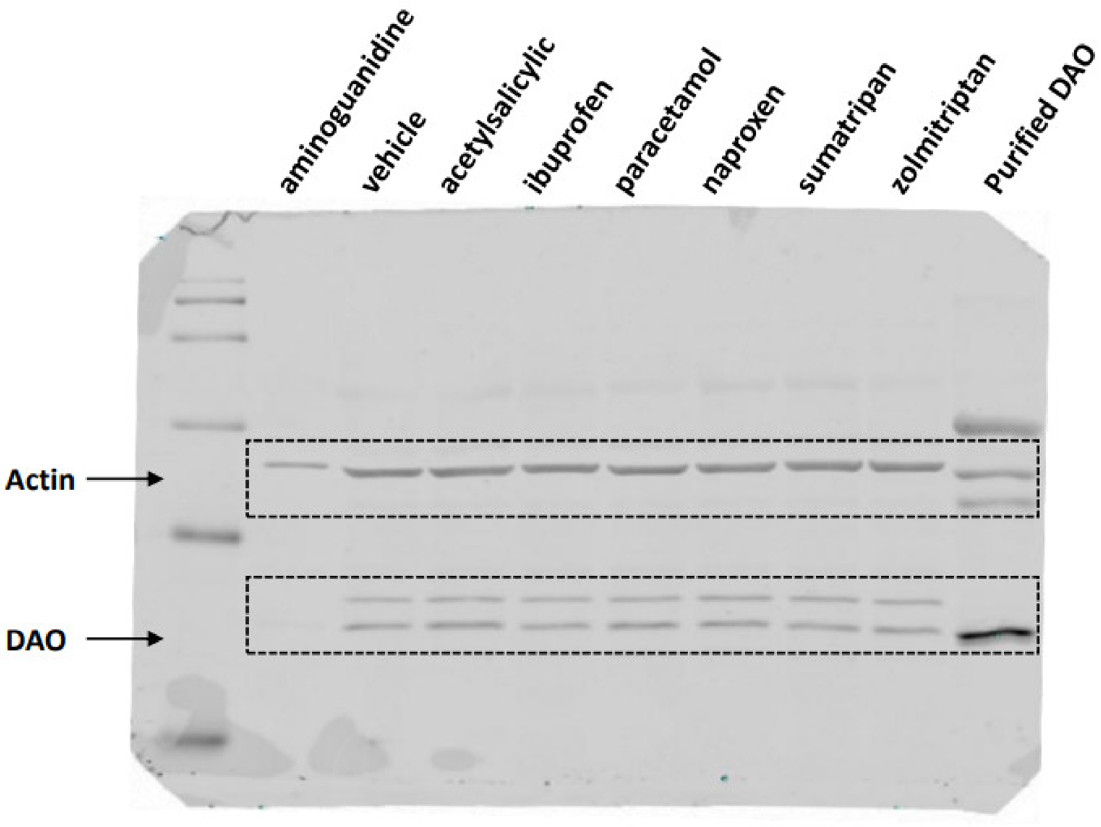

Figure S1. The analysis of DAO protein levels using Western Blot, without being cut. Furthermore, the levels of actin are displayed as the housekeeping gene, along with the different treatments corresponding to each well. The detection of actin and DAO is indicated with black arrows.
